# The Challenge of t (6;9) and FLT3-Positive Acute Myelogenous Leukemia in a Young Adult

**DOI:** 10.4172/2329-6917.1000167

**Published:** 2014-11-20

**Authors:** Yeohan Song, Dale Bixby, Diane Roulston, John Magenau, Sung Won Choi

**Affiliations:** 1 University of Michigan Medical School, University of Michigan, Ann Arbor, MI, USA; 2 Department of Internal Medicine, Division of Hematology-Oncology, University of Michigan, Ann Arbor, MI, USA; 3 Department of Pathology, University of Michigan, Ann Arbor, MI, USA; 4 Department of Pediatrics, Division of Pediatric Hematology-Oncology, University of Michigan, Ann Arbor, MI, USA

**Keywords:** Acute myelogenous leukemia, Translocation t(6;9), FLT3, Sorafenib, Trisomy 8

## Abstract

Translocation t(6;9) is a rare cytogenetic abnormality found in fewer than 5% of pediatric and adult cases of acute myelogenous leukemia (AML). The outcomes of t(6;9) AML are generally poor, with low five-year overall survival and increased risk for relapse. Furthermore, FLT3-ITD is one of the most common molecular abnormalities found in AML that is associated with increased risk of treatment failure and mortality. Allogeneic hematopoietic cell transplantation (HCT) with the best available donor is a standard treatment option for these cases once remission is achieved.

We report a challenging case of t(6;9) and FLT3-positive AML in a young adult male. After failing multiple standard induction regimens, morphologic remission was eventually achieved with a FLT3 inhibitor (sorafenib) and a hypomethylating agent (azacytidine). However, despite allogeneic HCT and re-initiation of sorafenib in the post-HCT setting, he experienced early relapse with the original [FLT3-ITD and t(6;9)] and new (FLT3-D835 and +8) molecular and cytogenetic markers, respectively. This case highlights the need for improved strategies in the post-HCT setting for high-risk AML.

## Background

Disease relapse after allogeneic hematopoietic cell transplantation (HCT) for acute myelogenous leukemia (AML) is the most frequent cause of post-transplantation treatment failure and mortality [1]. Management of relapse, particularly in the early post-transplantation setting, remains a significant challenge. While improved understanding of molecular and cytogenetic markers has led to the use of novel non-cytotoxic agents, there is no consensus on an optimal approach to post-transplantation maintenance therapy to improve outcomes of relapsed patients. Herein, we describe a case of highly refractory AML with recurrent cytogenetic abnormality t(6;9) and the FMS-like tyrosine kinase 3 gene (FLT3) internal tandem duplication (ITD) mutation and highlight the need for improved strategies to harness the immunotherapeutic benefits of HCT in conjunction with targetable agents.

## Case Presentation

A 23-year-old Caucasian male presented to his primary care physician with fever, night sweats, and bilateral lower extremity rash. Laboratory testing revealed white blood cells (WBC) 8.5×10^3^/μL (normal 4.0–10.0×10^3^), hemoglobin (Hgb) 10.4 g/dL (normal 13.5–17.0 g/dL), and platelets 41.0×10^3^/μL (normal 150–400×10^3^), with a manual differential count showing 66% blasts, 10% neutrophils, 1% myelocytes, 1% bands, and 18% lymphocytes. Bone marrow biopsy was significant for 74% blasts with Auer rods admixed with rare maturing granulocytic precursors. Flow cytometry detected an abnormal immature myeloid blast population with an immunophenotype of CD13, CD33, CD38, CD117, and HLA-DR. These cells also expressed low levels of CD4 and CD45; partially expressed CD7, CD25, and CD64; and variably expressed CD34. They did not express CD2, CD14, CD15, and CD56. Cytogenetic and molecular testing confirmed a diagnosis of AML with karyotype 46,XY,t(6;9)(p23;q34), DEK/NUP214 and the presence of the FLT3-ITD mutation, respectively. A skin biopsy of one of his lower extremity skin lesions was consistent with leukemia cutis.

**Figure 1:**
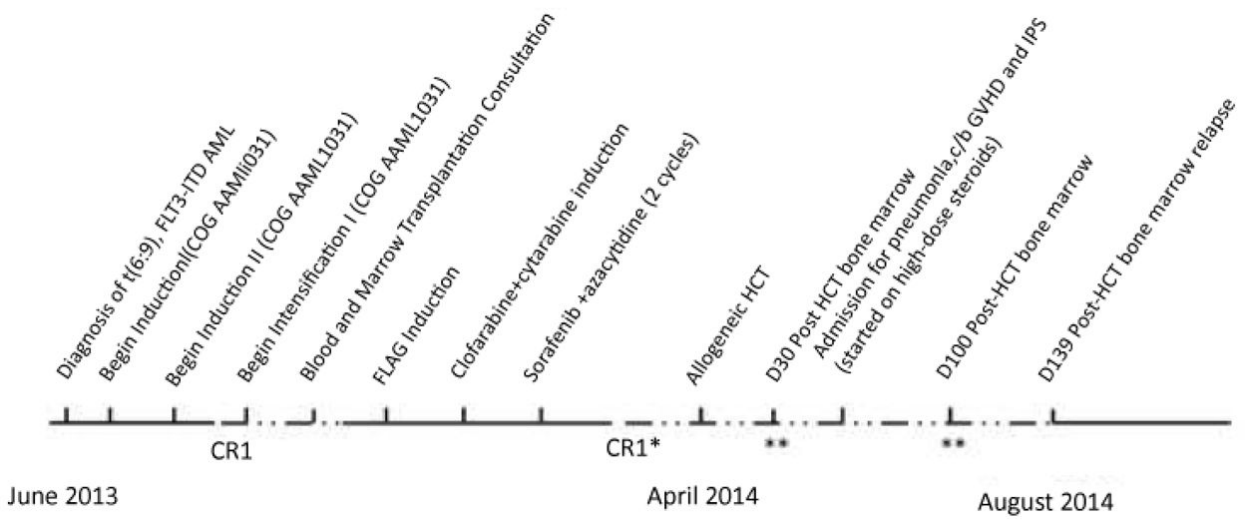
Diagram of patient’s treatment course. FLT3: FMS-Like Tyrosine Kinase 3 Gene; ITD: Internal Tandem Duplication; AML: Acute Myelogenous Leukemia; COG: Children’s Oncology Group; FLAG: Fludarabine, Cytarabine, Filgrastim; HCT: Hematopoietic Cell Transplantation; D: Day; GVHD: Graft-Versus-Host Disease; IPS: Idiopathic Pulmonary Syndrome; C/B: Complicated By Solid Line: Persistent or Recurrent Acute Myelogenous Leukemia (AML). Dotted Line: AML In Remission. *Morphologic remission, with persistent t(6;9) and FLT3-ITD. **Morphologic, flow cytometric, and molecular remission.

The patient consented to Children’s Oncology Group (COG) protocol AAML1031 treatment arm C, which included sorafenib, but was moved to treatment arm B, as sorafenib was on a temporary hold for toxicity observation (Figure 1). The induction treatment regimen consisted of 70 mg cytarabine (Ara-C) intrathecal (IT) on day 1; cytarabine 100 mg/m^2^ intravenous (IV) days 1–10; daunorubicin 50 mg/m2 days 1, 3, and 5; etoposide 100 mg/m^2^ days 1–5; and bortezomib 1.3 mg/m^2^ IV days 1, 4, and 8. Repeat bone marrow biopsy on day 25 of Induction I demonstrated 2% myeloblasts by morphology, consistent with the pre-treatment leukemia phenotype. A separate sample evaluated by the COG revealed the presence of 2.8% minimal residual disease (MRD), and fluorescence in situ hybridization (FISH) assay performed at Mayo Medical Laboratories (Rochester, MN) was positive for t(6;9) in 1.6% of nuclei.

The patient proceeded with Induction II, which included Ara-C 70 mg IT on day 5; cytarabine 1000 mg/m^2^ IV days 1–4; mitoxantrone 12 mg/m^2^ days 3–6; and bortezomib 1.3 mg/m2 IV days 1, 4, and 8. His course was complicated by febrile neutropenia, *Klebsiella* and *Escherichia coli* bacteremia, pericardial effusion, cellulitis, and a left upper extremity deep venous thrombosis. Repeat bone marrow biopsy on day 31 of Induction II demonstrated trilineage hematopoiesis with no morphologic, flow cytometric, or cytogenetic evidence of leukemia. FISH analysis was also negative for t(6;9), thus indicating first complete remission (CR1).

The patient started Intensification I two months after his initial diagnosis with Ara-C 70 mg IT on day 1; cytarabine 1000 mg/m^2^ IV days 1–5; etoposide 150 mg/m^2^ days 1–5; and bortezomib 1.3 mg/m2 IV days 1, 4, and 8. He was then referred to our Blood and Marrow Transplantation Team for consultation, and allogeneic HCT with the best possible donor was recommended.

Given the concerns of slow count recovery following Intensification I, repeat bone marrow biopsy was performed, which revealed 12% blasts. He was reinduced with fludarabine 30 mg/m^2^ IV days 1–5, cytarabine 2000 mg/m^2^ IV days 1–5, and filgrastim 5 mcg/kg starting day 1 (FLAG). Repeat bone marrow biopsy two weeks later revealed persistent AML with 25% blasts and t(6;9), with WBC 0.9×10^3^/μL, Hgb 9.6 g/dL, and platelets 23×10^3^/μL. Another reinduction regimen of clofarabine 40 mg/m2 IV days 2–6 and cytarabine 1000 mg/m^2^ IV days 1–5 was administered. However, repeat bone marrow biopsy showed persistent AML with 17% blasts, and cytogenetics confirmed karyotype 46,XY,t(6;9). The patient was referred to another hematologist to discuss alternative treatment options. Sorafenib 400 mg twice daily days 1–28 and azacytidine 75 mg/m^2^ days 1–7 was recommended. After two courses, the patient achieved a morphologic remission with negative flow cytometry, but demonstrated persistent cytogenetic and molecular positivity. MRD analysis sent to Hematologics, Inc. (Seattle, WA) was inconclusive due to ANC<1000.

The patient proceeded with a 9 of 10 HLA matched (HLA-B mismatched) unrelated donor peripheral blood HCT. The conditioning regimen consisted of fludarabine 40 mg/m^2^ IV and busulfan 3.2 mg/kg days −5 to −2, with the addition of thymoglobulin 2.5 mg/kg days −3 to −1 for mismatched HCT [2]. Body mass index was 31.4 kg/m^2^. The patient’s Hematopoietic Cell Transplantation-Specific Comorbidity Index (HCT-CI) score was 5, placing him in a high risk category. Graft versus host disease (GVHD) prophylaxis consisted of tacrolimus 0.03 mg/kg (starting day-3) and methotrexate 5 mg/m^2^ (days 1, 3, 6, 11). A cell dose of 5.6×10^6^ CD34 cells/kg was administered.

His clinical course was complicated by coagulase negative Staphylococcus central line infection, mucositis, deep venous thrombosis, and Clostridium difficile gastrointestinal infection. He engrafted neutrophils on day 11 with an absolute neutrophil count (ANC) of 0.6×10^3^/μL (>500 ANC on first of three consecutive days) and platelets on day 12 with a platelet count of 27×10^3^/μL (>20×10^3^/μL on first of three consecutive days). The patient was discharged on day 20. Day 30 bone marrow confirmed morphologic, flow cytometric, and molecular remission. Chimerism studies revealed 100% donor cells with CD3 and CD33, and MRD sent to Hematologics, Inc. was negative. The patient did well until day 36, when he was admitted for rhinovirus and Saccharomyces cerevisiae pneumonia and pericarditis. On day 44, he developed worsening respiratory symptoms requiring 2 L/min of supplemental oxygen, combined with progressively intense skin changes involving his hands and feet concerning for acute GVHD (not biopsy proven). The patient was initiated on prednisone 2 mg/kg/day (total dose 96 mg twice daily) for concern of idiopathic pulmonary syndrome (IPS). His respiratory symptoms resolved, and he was subsequently discharged on day 49.

**Figure 2:**
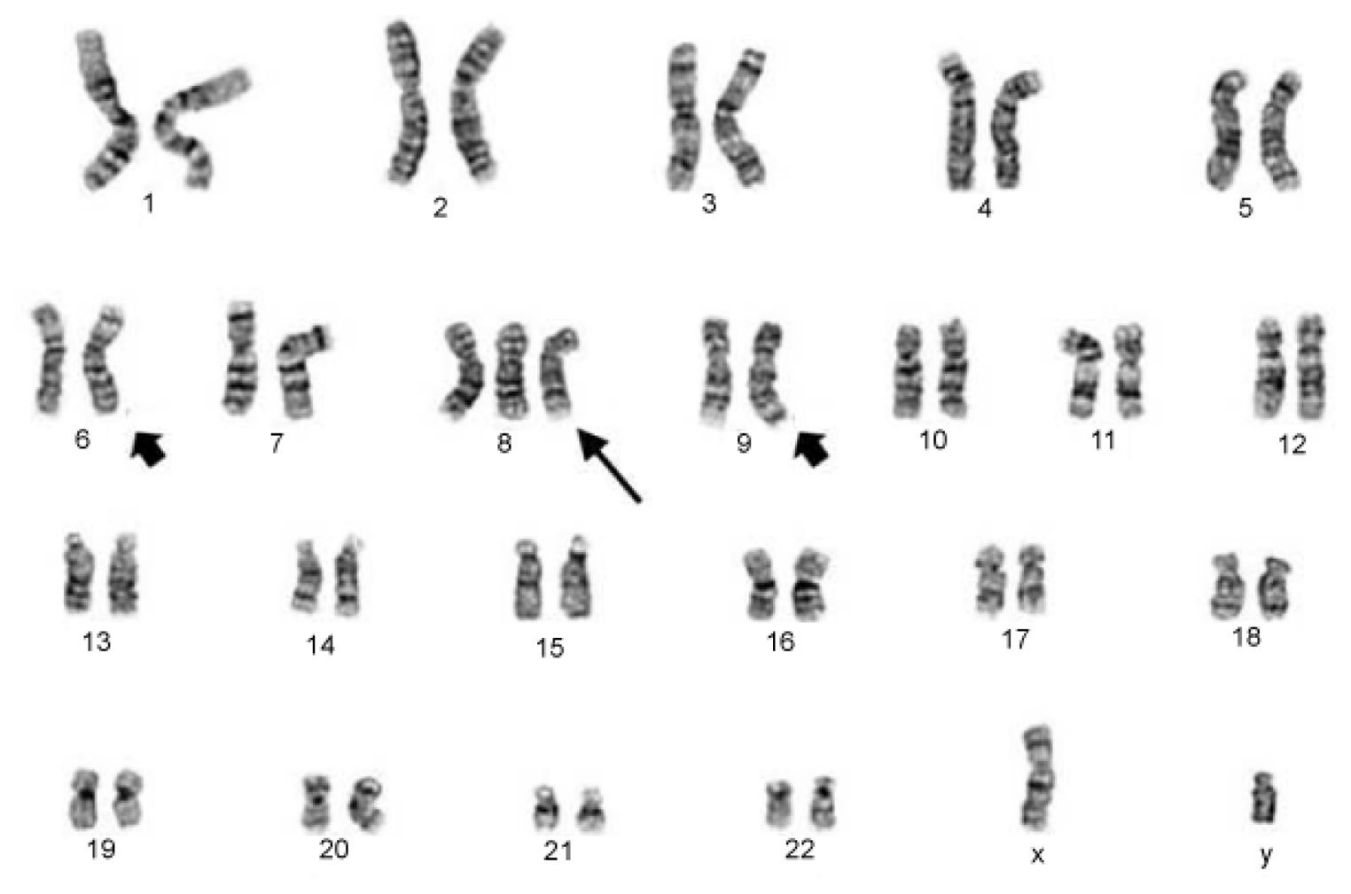
Post-transplantation day 139 karyogram showing 363 gain of chromosome 8 (long arrow) in addition to the previously identified t(6;9) chromosomal aberrancy (short arrows). International System for Human Cytogenetic Nomenclature (ISCN): 47,XY,t(6;9)(p23;q34),+8 [20]

Sorafenib was initiated on day 51, with a brief hold between day 65 and day 96 due to concern for thrombocytopenia (56–81×10^3^/μL). Repeat bone marrow testing on day 100 again showed no morphologic, flow cytometric, cytogenetic, or molecular evidence of AML. However, routine blood count monitoring again showed declining platelet counts (58×10^3^/μL) on day 132. Sorafenib was again placed on hold, and viral studies sent to determine the etiology of his thrombocytopenia were negative. Pathology review of the peripheral blood revealed blasts consistent with his previous leukemia, and a day 139 bone marrow confirmed relapsed disease. Molecular studies revealed new FLT3-D835 mutation in addition to previously detected FLT3-ITD. Cytogenetic testing confirmed presence of the previously identified t(6;9) and demonstrated a concomitant gain of chromosome 8 in all 20 metaphases analyzed (Figure 2). Chimerism studies showed 100% donor CD3 and 45% recipient CD33. Therapy options were discussed with the patient, and he is currently undergoing treatment with crenolanib as part of a phase II clinical trial (NCT01657682) for FLT3-positive relapsed AML.

## Discussion

Recent efforts have been made to identify prognostic factors for relapse risk (RR) in AML to direct more intensive treatment strategies to higher risk patients through a risk- adapted therapy approach [3]. FLT3-ITD is one of the most common abnormalities in AML, detected in 15 to 25% of patients, and a high ITD allelic ratio has been associated with five-year overall survival (OS) rates <35% and RR >60% [4–6]. Furthermore, AML patients positive for FLT3-ITD have been associated with increased rates of treatment failure and death even after achieving remission [6]. The human FLT3 gene encodes a cell surface protein found predominantly on hematopoietic progenitors and dendritic cells, and its activation leads to proliferation and survival responses normally regulated by tyrosine phosphorylation and proteasomal degradation [7,8]. However, in cases of FLT3-ITD with high ITD-AR, the FLT3 receptor remains constitutively active and leads to deregulation of downstream signaling. Allogeneic HCT is the only potentially curative therapy for AML with FLT3 activating mutations [9]. The role of instituting FLT3 inhibitors, such as sorafenib, in the post-HCT setting remains to be further explored [10,11].

The translocation t(6;9)(p23;q34) represents a rare subset of AML (<5% of adult and pediatric cases [12]) that is closely associated with FLT3-ITD [13,14] and is independently associated with lower five-year OS (≤40%) and higher RR (>50%) [15,16]. Patients with t(6;9) AML demonstrate lower response rates to induction chemotherapy and higher relapse rates despite achieving remission [15]. This translocation results in the formation of chimeric fusion gene DEK-NUP214 on the der(6) chromosome, which is translated to nucleoporin fusion proteins and leads to altered nuclear transport and upregulated myeloid protein synthesis [17,18]. This chromosomal aberrancy has been identified in both de novo and treatment-related AML [16,19]. Of note, the immunophenotype of our patient’s leukemic cells was consistent with that described (i.e., CD13+, CD33+, CD38+, CD45+, and HLA-DR+) [16].

This report highlights the challenge of managing AML with t(6;9) and FLT3-ITD positivity, which portend poor prognosis [4–6,15,16]. Our patient’s high-risk disease left him with few available treatment options following failure of four attempts at induction with standard chemotherapy regimens. However, among ongoing studies of investigative regimens is a phase 2 clinical trial using combination therapy with sorafenib, a FLT3 inhibitor, and azacytidine, a hypomethylating agent [20]. This combination is thought to promote lower levels of FLT3-ligand compared to standard chemotherapeutics by lessening the FLT3 ligand elevation associated with chemotherapy-induced aplasia [21,22]. A recent publication describes the results of this combination therapy for high-risk AML patients with FLT3-ITD who received a median of two prior treatment regimens, reporting a response rate of 46% and a complete response rate of 16% following a median of two cycles [20]. Indeed, we observed morphologic remission following two courses, consistent with the literature [20]. In efforts to minimize the risk for relapse [23], we continued post-HCT treatment with sorafenib.

Despite this, the patient experienced early post-transplantation relapse at day 139. Although remission was documented with the absence of morphologic, cytogenetic, and molecular features as far as day 100 post-transplantation, this high-risk disease recurred with not only the originally identified genetic abnormalities, but with the further addition of FLT3-D835 and trisomy 8 (+8). This is consistent with the major modes of clonal evolution demonstrated in AML: 1) the founding clone gains mutations following induction chemotherapy and persists to become the relapse clone or 2) a subclone of the founding clone survives induction, acquires additional mutations, and expands at relapse [24]. Given the presence of both previous and new cytogenetic abnormalities in all metaphases analyzed at the time of relapse, this case likely represents the first form of clonal evolution, with clonal expansion following HCT. The importance of these mutations is manifold. The presence of the FLT3-D835 mutation may confer increased resistance to tyrosine kinase inhibitors and lower disease-free survival [25], and the presence of trisomy 8, when associated with other cytogenetic abnormalities, has been attributed with lower overall survival at lower peripheral blast counts [26].

The cytogenetic and molecular characteristics observed at relapse may reflect the accumulation of acquired treatment-related abnormalities. There is a known risk of developing therapy-related changes leading to AML following topoisomerase-targeting chemotherapy, characterized by a shorter latency period from as little as a few months from exposure to diagnosis [27]. Additionally, treatment with tyrosine kinase inhibitors has been associated with acquired chromosomal abnormalities in chronic myelogenous leukemia, with trisomy 8 being the most commonly detected cytogenetic abnormality following treatment with imatinib [28]. Furthermore, the FLT3-D835 mutation has been observed in refractory AML maintained on sorafenib monotherapy without allogeneic HCT [29].

A recent review of the literature has found mixed results with post-transplantation use of sorafenib, and the role of this agent in treating FLT3-ITD AML remains to be determined [30]. An international survey of patients with FLT3-ITD AML refractory to a median of three chemotherapy cycles with or without prior allogeneic transplantation undergoing sorafenib monotherapy suggested that allogeneic transplantation may have a synergistic role with sorafenib in inducing allo-immune responses, but further demonstrated that over a third of these patients develop sorafenib resistance after a median treatment duration of six to seven months [31]. Recent case studies have also shown sorafenib to have a potential role in enhancing graft-versus-leukemia effects [32,33] or inducing myeloid maturation [33].

The present case highlights the high risk associated with t(6;9) independent of the FLT3-ITD status [15,16]. Moreover, the use of FLT3-targeted therapy in the post-HCT setting did not appear to prevent relapse. Further investigation into the role of DEK-NUP214 is needed, which may lead to novel strategies targeting this fusion protein.

## Conclusion

Relapse remains a significant cause of mortality after allogeneic HCT for AML. While the incorporation of non-cytotoxic targeted post-transplantation maintenance agents is currently being considered, the type of drug, disease-specific characteristics, timing of initiation, dosing/schedule, and hold/re-start parameters for myelosuppression need to be better defined and studied in appropriate clinical trials to ensure uniform practices across institutions.

## Abbreviations

AML: Acute Myelogenous Leukemia
ITD: Internal Tandem Duplication
HCT: Hematopoietic Cell Transplantation
